# Body mass index, smoking behavior, and depression mediated the effects of schizophrenia on chronic obstructive pulmonary disease: trans-ethnic Mendelian-randomization analysis

**DOI:** 10.3389/fpsyt.2024.1405107

**Published:** 2024-05-22

**Authors:** Yao Ni, DaWei Zhang, Wenlong Tang, Liming Xiang, Xiaoding Cheng, Youqian Zhang, Yanyan Feng

**Affiliations:** ^1^ Department of Dermatovenereology, Chengdu Second People’s Hospital, Chengdu, Sichuan, China; ^2^ Department of Clinical Laboratory, Chengdu Second People’s Hospital, Chengdu, Sichuan, China; ^3^ Department of Clinical Laboratory, The Second Affiliated Hospital of Chongqing Medical University, Chongqing, China; ^4^ Health Science Center, Yangtze University, Jingzhou, Hubei, China

**Keywords:** Mendelian randomization, chronic obstructive pulmonary disease, schizophrenia, mediation analysis, causality

## Abstract

**Background:**

Previous studies have highlighted the association between schizophrenia (SCZ) and chronic obstructive pulmonary disease (COPD), yet the causal relationship remains unestablished.

**Methods:**

Under the genome-wide significance threshold (*P*<5×10–^8^), data from individuals of European (EUR) and East Asian (EAS) ancestries with SCZ were selected for analysis. Univariable Mendelian randomization (MR) explored the causal relationship between SCZ and COPD. Linkage disequilibrium score (LDSC) regression was used to calculate genetic correlation, while multivariable and mediation MR further investigated the roles of six confounding factors and their mediating effects. The primary method utilized was inverse-variance weighted (IVW), complemented by a series of sensitivity analyses and false discovery rate (FDR) correction.

**Results:**

LDSC analysis revealed a significant genetic correlation between SCZ and COPD within EUR ancestry (r_g_ = 0.141, *P* = 6.16×10–^7^), with no such correlation found in EAS ancestry. IVW indicated a significant causal relationship between SCZ and COPD in EUR ancestry (OR = 1.042, 95% CI 1.013–1.071, *P* = 0.003, *P_FDR_* = 0.015). Additionally, replication datasets provide evidence of consistent causal associations(*P* < 0.05 & *P_FDR_* < 0.05). Multivariable and mediation MR analyses identified body mass index (BMI)(Mediation effect: 50.57%, *P* = 0.02), age of smoking initiation (Mediation effect: 27.42%, *P* = 0.02), and major depressive disorder (MDD) (Mediation effect: 60.45%, *P* = 6.98×10–^5^) as partial mediators of this causal relationship. No causal associations were observed in EAS (OR = 0.971, 95% CI 0.875–1.073, *P* = 0.571, *P_FDR_* = 0.761) ancestry. No causal associations were found in the reverse analysis across the four ancestries (*P* > 0.05 & *P_FDR_* > 0.05).

**Conclusions:**

This study confirmed a causal relationship between SCZ and the risk of COPD in EUR ancestry, with BMI, smoking, and MDD serving as key mediators. Future research on a larger scale is necessary to validate the generalizability of these findings across other ancestries.

## Introduction

Chronic obstructive pulmonary disease (COPD) is a prevalent respiratory disorder characterized by persistent respiratory symptoms and airflow limitation (1). Globally, COPD affects approximately 540 million people, ranking as the third leading cause of death and the seventh leading cause of health impairment (2). It often results in psychological conditions such as anxiety and depression in patients, imposing significant burdens on healthcare systems and the economy (1). With population aging and environmental factors, the incidence of COPD is projected to continue rising over the next 40 years (3). Prevention of COPD is particularly challenging due to its complex etiology involving genetic, environmental, and lifestyle factors. Therefore, identifying and understanding modifiable risk factors for COPD is crucial for its prevention and management.

Clinically, COPD patients are often accompanied by psychiatric disorders or psychological disorders, suggesting that COPD should no longer be regarded as a pulmonary disease (4).

Studies have shown that patients with serious psychiatric disorders such as schizophrenia (SCZ) or bipolar disorder are at increased risk for physical comorbidities, including respiratory disorders (5). A systematic review reported a prevalence of COPD in patients with SCZ ranging from 2.6% to 52.7% (6). A Taiwanese study showed that patients with SCZ had a higher prevalence of COPD than the general population. Compared with the general population, patients with SCZ have a higher prevalence of COPD in those younger than 50 years and those older than 70 years (7). Patients with SCZ have an increased risk of COPD, which may be related to the significantly lower lung function values of patients with SCZ (8). However, there is still a lack of genetic evidence for SCZ and COPD population distribution, confounding factors, and reverse causality studies.

However, traditional epidemiological research is inherently flawed, often limited by its inability to eliminate confounding factors and establish causal relationships. Although randomized controlled trials (RCTs) are the "gold standard" for establishing causality, large-scale RCTs are often hindered by high costs and significant time and resource requirements. In this context, Mendelian randomization (MR) analysis emerges as a viable alternative to RCTs. MR employs genetic variants identified in Genome-Wide Association Studies (GWAS) as instrumental variables (IVs) for determining the causal effects of risk factors, or exposures, on outcomes (9). Owing to the random allocation of alleles at birth, MR adeptly addresses confounders and reverse causation, which are prevalent challenges in traditional epidemiological studies (10). Although previous MR studies have suggested that there is no association between COPD and SCZ, these studies were limited to European populations, and no detailed study of confounding and mediating factors was performed, so the results of MR studies of COPD and SCZ are still questionable (11). Given the complexity and many confounding factors in the pathogenesis of COPD, this study aimed to explore the potential causal relationship between SCZ and COPD using MR analysis to provide new insights and evidence on this important clinical issue.

## Materials and methods

### Study design

**Figure 1:**
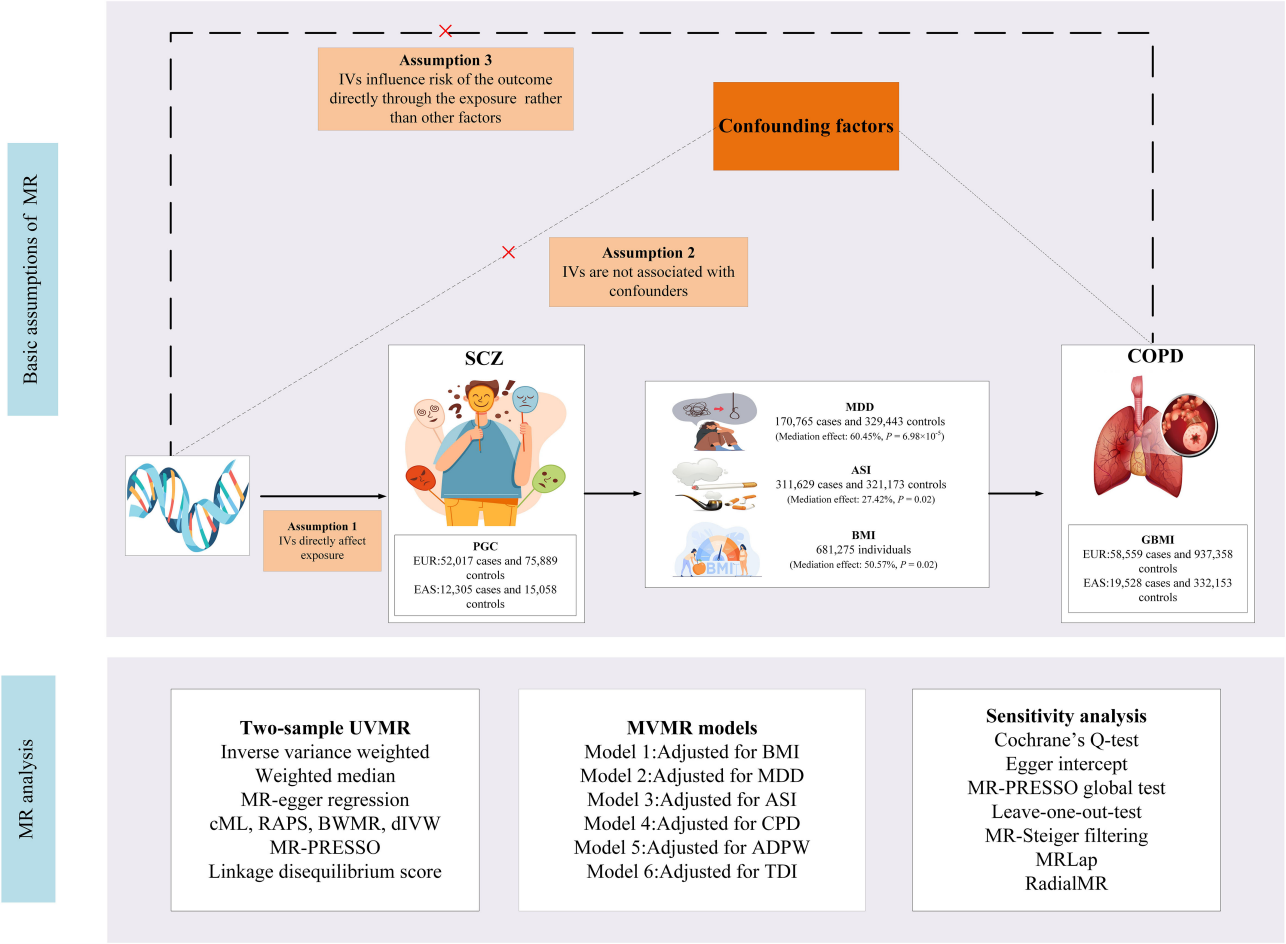
Study design. SCZ, Schizophrenia; COPD, chronic obstructive pulmonary disease; BMI, body mass index; GSCAN, GWAS and Sequencing Consortium of Alcohol and Nicotine use; GIANT, Genetic Investigation of Anthropometric Traits; PGC, Psychiatric Genomics Consortium; CPD, cigarettes per day; ASI, age of smoking initiation; ADPW, Alcoholic drinks per week; TDI, Townsend deprivation index; GBMI, Global Biobank Meta-analysis Initiative; EUR, European; EAS, East Asian; MDD, Major depression disorder; MR-PRESSO, MR Pleiotropy Residual Sum and Outlier; IVW, inverse-variance-weighted; RAPS, robust adjusted profile score; CML, constrained maximum likelihood; dIVW, debiased inverse-variance weighted; SNP, single nucleotide polymorphism; IV, instrumental variable; MR, Mendelian randomization; UVMR, univariable MR; MVMR, multivariable MR; BWMR, Bayesian weighted Mendelian randomization.

This study employed primary datasets from publicly available GWAS to explore the putative causal relationships between specific exposures and outcomes. We adopted a range of sophisticated analytical methodologies, including univariable MR (UVMR), multivariable MR (MVMR), genetic correlation, and mediation MR analysis. The selection of IVs for these exposures adhered to three essential criteria: (i) the selected genetic markers, functioning as IVs, demonstrate a robust association with the exposure; (ii) these genetic markers are not linked to potential confounding variables; and (iii) the influence of the genetic variants on the outcome is exclusively mediated via the exposure, excluding alternative pathways (12). A detailed representation of the MR methodology is illustrated in **Figure 1**. Comprehensive summary statistics derived from these datasets are systematically presented in **Table 1**.

**Table 1:** Detailed information of data sources.

Phenotype	Ref	Ieu id	Consortium	Ancestry	Participants
Phenotypes					
SCZ	35396580	ieu-b-5102	PGC	EUR	52,017 cases and 75,889 controls
SCZ	35396580	ieu-b-5101	PGC	EAS	12,305 cases and 15,058 controls
COPD (Discovery Dataset)	36777996	NA	GBMI	EUR	58,559 cases and 937,358 controls
COPD (Replication Dataset)	36653562	NA	FinnGen	EUR	20,066 cases and 338,303 controls
COPD (Discovery Dataset)	36777996	NA	GBMI	EAS	19,528 cases and 332,153 controls
COPD (Replication Dataset)	34594039	ebi-a-GCST90018587	Sakaue S	EAS	4,017 cases and 162,153 controls
Adjustment of the model					
MDD	30718901	ieu-b-102	PGC	EUR	170,765 cases and 329,443 controls
ASI	30643251	ieu-b-4877	GSCAN	EUR	311,629 cases and 321,173 controls
CPD	30643251	ieu-b-25	GSCAN	EUR	337,334 individuals
ADPW	30643251	ieu-b-73	GSCAN	EUR	335,394 individuals
BMI	30124842	ieu-b-40	GIANT	EUR	681,275 individuals
TDI	34017140	ebi-a-GCST90013973	Mbatchou J	EUR	407,271 individuals

SCZ, Schizophrenia; COPD, chronic obstructive pulmonary disease; BMI, body mass index; GSCAN, GWAS and Sequencing Consortium of Alcohol and Nicotine use; GIANT, Genetic Investigation of Anthropometric Traits; PGC, Psychiatric Genomics Consortium; CPD, cigarettes per day; ASI, age of smoking initiation; ADPW, Alcoholic drinks per week; TDI, Townsend deprivation index; GBMI, Global Biobank Meta-analysis Initiative; EUR, European; EAS, East Asian; MDD, Major depression disorder; NA, not applicable.

### Selection of genetic instrumental variables

To ensure rigorous and accurate assessments, our study established stringent criteria for the selection of single nucleotide polymorphisms (SNPs) as IVs:

- SNPs identified as IVs were required to demonstrate a genome-wide significant association with the specified exposure (*P* < 5×10^–8^).
- Selected SNPs were subjected to thorough scrutiny to eliminate links with potential confounders and to ascertain their distinctiveness, thus minimizing biases arising from linkage disequilibrium (LD). This involved setting stringent LD parameters (1000 Genomes Project Phase 3: r^2^ < 0.001, clumping distance = 10,000 kb). The LDTrait tool was used to identify traits (https://ldlink.nih.gov/?tab=home), ensuring adherence to the assumption of independence (13). Based on previous experience with MR analyses (14), SNPs associated with smoking, lung function, emphysema, asthma, obesity, and body mass index (BMI) were excluded.
- The appropriateness of SNPs as IVs was appraised using F-statistics (F = beta²/se², where beta represents the SNP-exposure association strength and se denotes the standard error) (15). This evaluation aimed to identify and exclude weak instrumental variables, with an F-statistic threshold above 10 being a prerequisite for SNP inclusion.
- To enhance the validity of our findings, MR-Steiger filtering was implemented, excluding any variants that exhibited a stronger correlation with the outcome than with the exposure (*P* < 5×10–^5^) (16).
- To ensure the accuracy of the results, proxy SNPs (r^2^ > 0.8) were not utilized in this study.
- A fundamental requirement for SNP selection was that its effects on both the exposure and outcome should be consistent with the same allele direction.

### Source of schizophrenia phenotype

The most comprehensive and recent GWAS data on SCZ originates from the Psychiatric Genomics Consortium (PGC) (17), spearheaded by Vassily Trubetskoy and his team. This cross-ethnic meta-analysis amalgamated individual genotypes from 90 European (EUR) and East Asian (EAS) ancestry cohorts within the core PGC dataset, encompassing 52,017 cases and 75,889 controls of EUR ancestry, and 12,305 cases and 15,058 controls of EAS ancestry. The primary GWAS identified 313 independent SNPs with LD r^2^ < 0.1, surpassing genome-wide significance (*P* < 5×10–^8^).

### Source of chronic obstructive pulmonary disease phenotype

The outcome phenotype COPD data were sourced from the Global Biobank Meta-analysis Initiative (GBMI) (18). As of November 2023, the GBMI has amalgamated a collaborative network comprising 24 biobanks from 15 countries across four continents, representing over 2.2 million consenting individuals and approximately 70 million genetic variants. The GBMI provides the most comprehensive multi-ancestry GWAS data on COPD to date, encompassing GWAS summary data for EUR ancestry (58,559 cases and 937,358 controls), and EAS ancestry (19,528 cases and 332,153 controls). These data were derived from meta-analyses integrating cohorts from 12 and 4 biobanks, respectively. To mitigate the risk of bias associated with reliance on a single dataset, further validation was conducted using supplementary replication datasets. For individuals of EUR ancestry, data were sourced from the latest R10 release of the FinnGen consortium (20,066 cases and 338,303 controls) (19). Similarly, for individuals of EAS ancestry, data were derived from a meta-analysis conducted by Sakaue et al. (4,017 cases and 162,153 controls) (20).

### Data sources for possible confounders

The study further obtained genetic associations for BMI from the Genetic Investigation of Anthropometric Traits (GIANT) consortium (21), cigarettes per day (CPD), age of smoking initiation(ASI), and alcoholic drinks per week (ADPW) from GWAS and Sequencing Consortium of Alcohol and Nicotine use (GSCAN) (22), major depression disorder (MDD) from PGC (23), townsend deprivation index (TDI) from Mbatchou J (24).

### Statistical analyses

#### MR analysis

In our MR analysis, the UVMR framework employed the Wald ratio test to evaluate each IV. Concurrently, the multiplicative random-effects inverse-variance-weighted (IVW) method was utilized to establish causal connections across multiple IVs (≥2). This approach was further augmented by both the MR-Egger and weighted median methods. In IVW, the weighting corresponds with the Wald ratio for each SNP and inversely correlates with its variance (25). The IVW method, considering a broad range of genetic variants, offers consistent and systematic results. The weighted median method is crucial when over half of the genetic variants are deemed invalid, whereas MR-Egger assumes the complete invalidity of these variants (26). To account for multiple comparisons in our analysis, we employed the False Discovery Rate (FDR) correction method. Post-FDR adjustment, a *P*-value of less than 0.05 is deemed indicative of a statistically significant causal relationship. Conversely, a P-value of less than 0.05 accompanied by an FDR-adjusted *P* -value greater than 0.05 is regarded as suggestive, but not conclusive, of such a relationship.

The study employed multiple methodologies to enhance the causal inference drawn from the primary analysis method, effectively mitigating biases associated with potential pleiotropy and confounding variables. Initially, Bayesian weighted Mendelian randomization (BWMR) addresses violations of IV assumptions due to pleiotropy and compensates for uncertainties stemming from weak genetic effects related to polygenicity, thereby strengthening the robustness of causal estimates (27). This approach utilizes a variational expectation-maximization (VEM) algorithm to stabilize and optimize the computation of causal inferences derived from BWMR. Furthermore, the robust adjusted profile score (RAPS) method refines the profile likelihood of the Wald ratio by adjusting the contribution of each IV, proving particularly effective in mitigating the impact of extreme outliers (28). This adjustment reduces the influence of anomalous IVs on the overall causal estimate, enhancing its reliability in scenarios complicated by pleiotropy. Additionally, the constrained maximum likelihood (cML) approach enabled comprehensive evaluations across various genetic variants, accounting for potential confounders and genetic variation. This method is particularly effective with a large array of genetic variants and confounders, providing precise and robust results (29). The debiased inverse-variance weighted (dIVW) method utilizes data from all IVs, making adjustments and corrections for biases in each SNP estimate arising from pleiotropy and weak IVs (30). Employed alongside other supplementary methods, the dIVW enhances the study’s causal inference, ensuring robustness in the analysis and diminishing biases associated with potential pleiotropy and confounding factors.

To elucidate the direct causal pathways from exposure to outcome, we performed additional MVMR analyses (31). These analyses aimed to rigorously define the direct causal links, distinguishing them from the UVMR model. Unlike UVMR, which focuses on a single exposure, MVMR accounts for genetic variations associated with multiple exposures. The initial phase of this analysis entailed obtaining MR effect estimates for exposure-to-outcome relationships using the IVW approach. This was followed by an MVMR analysis to assess the impact of six mediators on the outcome, with consideration of the characteristics of the exposure. To deduce the indirect effects of the exposure, we computed the derived estimates for each outcome. The final stage of this analysis involved calculating the ratio of the mediation effect to the total effect, providing insights into the relative contribution of these mediators to the overall outcome.

### LDSC regression analysis

Linkage Disequilibrium Score (LDSC) regression, designed for GWAS summary data, explores genetic correlations across complex traits by leveraging linkage disequilibrium from the 1000 Genomes Project Phase 3. This panel includes diverse ancestries such as EUR and EAS, ensuring accurate genetic diversity representation (32). LDSC distinguishes genuine polygenic effects from confounders like population stratification. In our study, it was used to analyze genetic relationships between SCZ and COPD, showing how genetic correlations extend beyond environmental influences. Detailed LDSC method settings are documented on the platform at https://github.com/bulik/ldsc, enhancing transparency and reproducibility.

### Sensitivity analysis

In the UVMR analysis, a range of methodological assessments were conducted. Cochran’s Q test was applied to assess heterogeneity among the selected genetic variants, with a P-value less than 0.05 indicating significant variability among the SNPs (33). The presence of directional pleiotropy within the MR framework was investigated using MR-Egger regression (34), where an intercept *P*-value below 0.05 suggests significant directional pleiotropy, within the method’s limitations (35). The MR Pleiotropy Residual Sum and Outlier (MR-PRESSO) method was used to identify potential outliers and evaluate horizontal pleiotropy, confirmed by a global *P*-value under 0.05 (36). Outliers were carefully excluded to refine our analysis, followed by a leave-one-out analysis, which assessed the impact of each SNP on the overall results (37). Moreover, MR-PRESSO has certain limitations, particularly when handling a large number of SNPs, as it may not effectively identify outliers to be excluded, potentially leading to unavoidable pleiotropy. Consequently, RadialMR serves as a valuable complement. RadialMR analysis generates radial plots that visually depict outliers, facilitating their thorough removal (38). However, the exclusion of a substantial number of SNPs could result in “blind noise reduction,” thereby diminishing the statistical power obtained (39). Therefore, the results from RadialMR analysis should be considered only for sensitivity analysis, to confirm whether the causal associations remain robust in the absence of heterogeneity and pleiotropy.

In the exposure and outcome datasets, while no evident sample overlap was detected, the possibility of latent sample overlap leading to the winner’s curse cannot be excluded. To address this, an MRLap analysis was conducted in the discovery dataset to correct for potential biases. MRLap analysis utilizes cross-trait LDSC intercepts to adjust for biases introduced by sample overlap and the winner’s curse, demonstrating good model fit within a 5%-95% sample overlap range (40). For each selected SNP, R^2^ values were calculated using the formula 2×MAF×(1-MAF)×beta^2^, where MAF denotes the minor allele frequency. These values were aggregated to compute the coefficient essential for determining statistical power (41). The mRnd website (39) (https://shiny.cnsgenomics.com/mRnd/) was employed to ascertain the statistical power of our analyses.

## Results

### Genetic instrument selection and genetic correlation between phenotypes

Scatter plots provided a clear visualization of the direction of causal relationships (**Supplementary Figure S1**). The IVs used in the analysis varied from 5 to 138, accounting for genetic variations ranging from 0.92% to 22.76%. Specifically, using LDTrait, three confounding SNPs were removed from the GBMI cohort within the EUR ancestry, while six were excluded from the FinnGen cohort. No confounding SNPs were removed in the EAS ancestry (**Supplementary Tables S1–S3**). All SNPs underwent MR-Steiger filtering with F-statistics exceeding 10, substantially reducing bias due to weak IVs. Detailed information for each SNP is presented in **Supplementary Tables S5–S9**.

LDSC analysis revealed a significant genetic correlation between SCZ and COPD in EUR ancestry (r_g_ = 0.141, *P* = 6.16×10–^7^), but no such correlation was found in EAS (r_g_ = -0.093, *P* = 0.23). The SNP-based liability-scale heritability (h²) varied from 0.75% to 44.52% (**Supplementary Table S10**).

### Association of genetically predicted exposure with outcome

**Figure 2:**
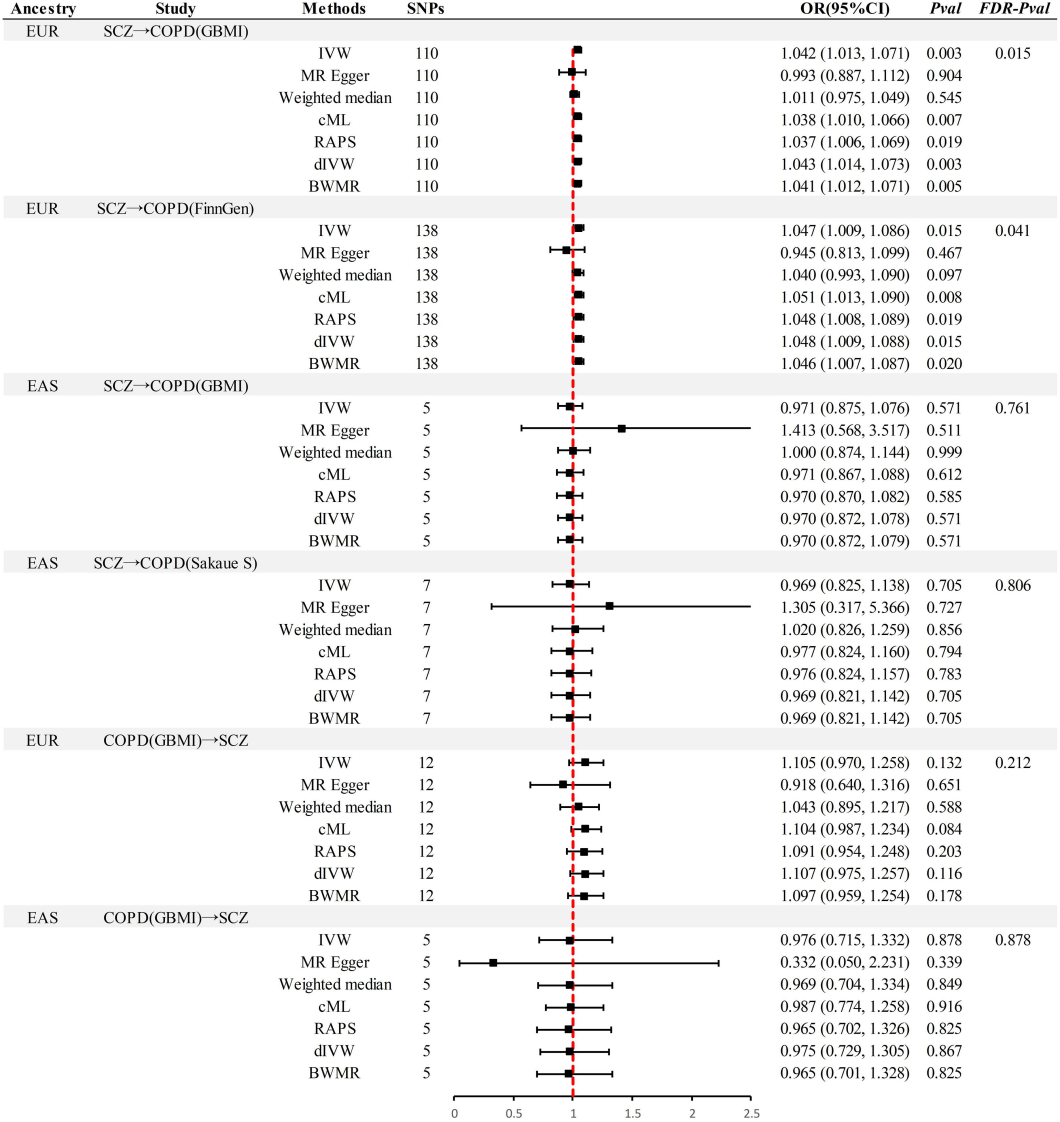
Summary of univariable Mendelian randomization analysis. SCZ, Schizophrenia; COPD, chronic obstructive pulmonary disease; GBMI, Global Biobank Meta-analysis Initiative; EUR, European; EAS, East Asian; IVW, inverse-variance-weighted; RAPS, robust adjusted profile score; CML, constrained maximum likelihood; dIVW, debiased inverse-variance weighted; SNP, single nucleotide polymorphism; IV, instrumental variable; MR, Mendelian randomization; OR, Odds Ratio; CI, Confidence Interval; BWMR, Bayesian weighted Mendelian randomization.

In the MR analysis (**Figure 2**), significant causal associations were identified in EUR ancestry after FDR correction. Specifically, the IVW method indicated that each standard deviation (SD) increase in genetically predicted SCZ was associated with a 4.2% increase in the incidence of COPD (Odds Ratio [OR] = 1.045, 95% Confidence Interval [CI] 1.013–1.071, *P* = 0.003, *P_FDR_* = 0.015). Supplementary methods including cML, RAPS, dIVW, and BWMR provided consistent causal evidence. Furthermore, the replication dataset corroborated the findings from the discovery dataset, affirming the robustness of the causal association(*P* < 0.05& *P_FDR_* < 0.05). When the OR are 1.042 and 1.047, we possess sufficient statistical power to detect the association between them, further strengthening the robustness of the causal evidence (Power > 99%) (**Supplementary Table S11**). Moreover, no additional causal evidence was found in analyses involving East Asian ancestry or reverse causation, with supplementary methods remaining consistent (*P* > 0.05& *P_FDR_* > 0.05).

### Sensitivity analyses

**Figure 3:**
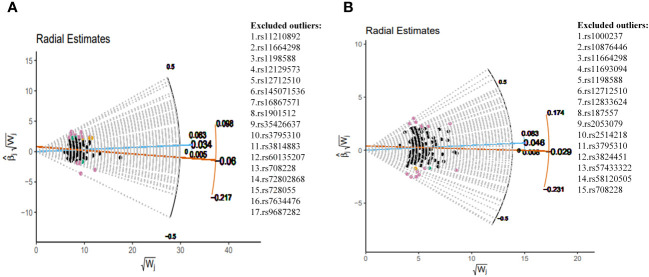
Complete exclusion of outliers by RadialMR-assisted MR-PRESSO in the EUR. **(A)** SCZ→COPD(GBMI), **(B)** SCZ→COPD(FinnGen). SCZ, Schizophrenia; COPD, chronic obstructive pulmonary disease; GBMI, Global Biobank Meta-analysis Initiative; EUR, European; MR-PRESSO, MR Pleiotropy Residual Sum and Outlier.

**Figure 4:**
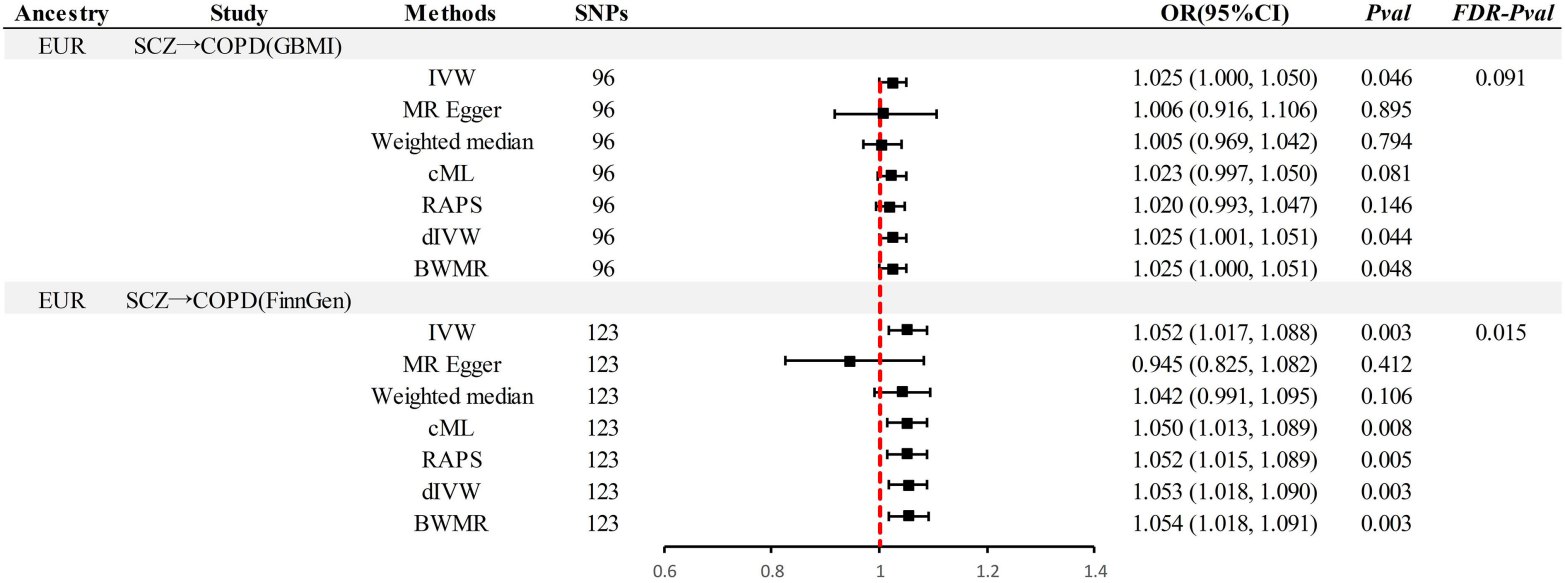
Results after thorough exclusion of outliers by RadialMR in the EUR pedigree. SCZ, Schizophrenia; COPD, chronic obstructive pulmonary disease; GBMI, Global Biobank Meta-analysis Initiative; EUR, European; IVW, inverse-variance-weighted; RAPS, robust adjusted profile score; CML, constrained maximum likelihood; dIVW, debiased inverse-variance weighted; SNP, single nucleotide polymorphism; MR, Mendelian randomization; OR, Odds Ratio; CI, Confidence Interval; BWMR, Bayesian weighted Mendelian randomization.

This study implemented several methods to validate the robustness of the causal associations. MRLap confirmed that the causal associations remain robust, unaffected by potential biases from sample overlap (**Supplementary Table S12**). MR-Egger testing detected no evidence of horizontal pleiotropy, and no heterogeneity or pleiotropy was observed in analyses involving East Asian ancestry or reverse causation (**Supplementary Table S13**). However, in the analysis of SCZ on COPD within EUR ancestry, unavoidable heterogeneity and pleiotropy arose due to an excess of IVs. Subsequent reanalysis, after excluding 17 and 15 outliers in the GBMI and FinnGen cohorts respectively via RadialMR (**Figure 3**), yielded results consistent with the original analyses (**Figure 4**). Nonetheless, the exclusion of a significant number of SNPs resulted in a reduction of statistical power (**Supplementary Table S11**). Leave-one-out analysis indicated that the derived causal associations were not driven by any single SNP, ensuring the robustness of the results (**Supplementary Figure S2**).

### Mediation and MVMR analysis

In the UVMR analysis, evidence supported a causal association between SCZ and the risk of COPD in EUR ancestry, achieving statistical significance (*P* < 0.05 & *P_FDR_* < 0.05). Further, MVMR analysis (**Table 2**), accounting for potential confounding phenotypes and adjustments for MDD (OR = 1.027, 95% CI 0.983–1.072, *P* = 0.23), ASI (OR = 1.032, 95% CI 0.995–1.070, *P* = 0.07), BMI (OR = 1.025, 95% CI 0.980–1.073, *P* = 0.28), TDI (OR = 1.036, 95% CI 0.998–1.076, *P* = 0.06), and all phenotypes (OR = 0.982, 95% CI 0.937–1.029, *P* = 0.44), indicated that the causal relationship between SCZ and COPD was no longer significant. This suggests that these confounding factors may partially mediate the causal link between SCZ and COPD. Subsequent mediation MR analysis revealed that TDI did not exhibit a mediating effect (**Table 3**), merely acting as a confounder, whereas MDD (Mediation effect: 60.45%, *P* = 6.98×10–^5^), ASI (Mediation effect: 27.42%, *P* = 0.02), and BMI (Mediation effect: 50.57%, *P* = 0.02) served as mediators, partially conveying the causal influence of SCZ on COPD.

**Table 2:** Adjustment for MVMR analysis in SCZ on COPD.

MVMR models	SNP	OR(95%CI)	*Pval*
MDD	130	1.347 (1.141, 1.589)	4.19E-04
SCZ		1.027 (0.983, 1.072)	0.230
ASI	152	1.568 (1.388, 1.771)	5.02E-13
SCZ		1.032 (0.995, 1.070)	0.089
CPD	120	1.541 (1.414, 1.681)	1.03E-22
SCZ		1.038 (1.003, 1.074)	0.031
ADPW	126	0.927 (0.644, 1.334)	0.683
SCZ		1.058 (1.018, 1.101)	0.005
BMI	362	1.453 (1.360, 1.553)	1.72E-28
SCZ		1.025 (0.980, 1.073)	0.279
TDI	116	3.338 (1.783, 6.248)	1.65E-04
SCZ		1.036 (0.998, 1.076)	0.064

SNP, single nucleotide polymorphism; MVMR, Multivariable Mendelian randomization; SCZ, Schizophrenia; COPD, chronic obstructive pulmonary disease; BMI, body mass index; CPD, cigarettes per day; ASI, age of smoking initiation; ADPW, Alcoholic drinks per week; TDI, Townsend deprivation index; OR, Odds Ratio; CI, Confidence Interval.

**Table 3:** Mediation analysis of the mediation effect of SCZ on COPD (GBMI-EUR) via six confounding factors.

Outcome	Mediator	Total effect Effect size (95% CI)	Direct effect Effect size (95% CI)	Mediation effect		
				Effect size (95% CI)	IE div TE(%)	*P*
COPD	MDD	0.034 (0.006, 0.063)	0.014 (-0.017, 0.044)	0.021 (0.010, 0.031)	60.45%	6.98E-05
	ASI	0.034 (0.006, 0.063)	0.025 (-0.005, 0.055)	0.009 (0.001, 0.018)	27.42%	0.02
	CPD	0.034 (0.006, 0.063)	0.026 (-0.005, 0.056)	0.008 (-0.002, 0.018)	24.64%	0.09
	ADPW	0.034 (0.006, 0.063)	0.032 (0.003, 0.061)	0.002 (-0.002, 0.006)	6.21%	0.26
	BMI	0.034 (0.006, 0.063)	0.017 (-0.015, 0.049)	0.017 (0.003, 0.032)	50.57%	0.02
	TDI	0.034 (0.006, 0.063)	0.037 (0.008, 0.067)	-0.003 (-0.009, 0.003)	-8.84%	0.33

BMI, body mass index; IE div TE,Indirect Effect divided by Total Effect; CI, confidence interval; SCZ, Schizophrenia; COPD, chronic obstructive pulmonary disease; CPD, cigarettes per day; ASI, age of smoking initiation; ADPW, Alcoholic drinks per week; TDI, Townsend deprivation index; MDD, Major depression disorder; GBMI, Global Biobank Meta-analysis Initiative; EUR, European.

## Discussion

This study conducted a comprehensive multi-ancestry MR analysis to investigate the causal relationship between the genetic susceptibility to SCZ and the risk of COPD. The MR findings corroborated previous epidemiological studies, establishing a causal link between genetically predicted SCZ and increased risk of COPD in European ancestry, including the mediating roles of BMI, ASI, and MDD. However, the study did not observe causal effects in other ancestries. We will discuss the implications of these findings from the perspectives of neuro-immune interactions, socio-economic and behavioral factors, mental and physiological health factors, and regional population differences.

SCZ is associated with alterations in immune system function. Imbalances in immune-regulatory metabolites in the serum of SCZ patients may contribute to systemic immune activation (42). This condition likely involves abnormal expression of various inflammatory mediators and cytokines, such as tumor necrosis factor (TNF) and interleukins (ILs), as consistent evidence provided by the review study of Rachel Upthegrove and colleagues indicates (43). These alterations result in a chronic state of systemic inflammation, leading to pulmonary tissue damage and diminished lung function, thereby impacting respiratory health and increasing the risk of COPD. Additionally, compromised immune function elevates the risk of respiratory infections, further exacerbating the development of COPD (44).

Patients with SCZ are often linked to socio-economic factors and unhealthy behaviors. Firstly, smoking is a primary risk factor for many respiratory diseases, notably COPD. The ASI can influence an individual’s susceptibility to tobacco exposure, with early initiation increasing the total lifetime exposure to tobacco, thereby elevating the risk of developing COPD. A MR study by Robyn E. Wootton’s team further provides evidence of a causal association between SCZ and increased tobacco consumption (45). Additionally, individuals with SCZ may experience elevated BMI and metabolic disorders due to factors such as lack of physical activity, poor dietary habits, or the disease itself (46). Numerous studies indicate that both smoking and obesity adversely affect lung function and exacerbate pulmonary inflammation, oxidative stress, and immune system dysfunction, thereby promoting the development of COPD (47–49). Furthermore, the Multivariable MVMR analysis in this study revealed that after adjusting for the TDI, the significant association between SCZ and COPD was lost (OR = 1.036, 95% CI 0.998–1.076, *P* = 0.06), underscoring the role of socio-economic status. Lifestyle choices are often influenced by an individual’s socio-economic status (SES), and an MR study by Jiahao Cai and colleagues highlights the association of low SES with an increased risk of SCZ (50). Economic hardship or social marginalization can lead to unhealthy dietary habits, high tobacco consumption, and lack of adequate medical care, thus increasing the risk of COPD. There is an interplay between lifestyle and socio-economic factors, which may be more complex in patients with SCZ, as mental health status itself can affect employment opportunities and economic conditions, thereby influencing lifestyle choices. Hence, the lifestyle and socio-economic status of SCZ patients together form a complex network contributing to their risk of COPD, necessitating comprehensive interventions to mitigate these risks.

Psychophysiological health factors play a significant role in the association between SCZ and COPD. The side effects of first-generation antipsychotic medications, such as chlorpromazine, fluphenazine, and thioridazine, can influence endocrine and metabolic processes, affecting hormonal balance and psychological states. Drug-induced side effects like weight gain and insulin resistance increase the risk of developing COPD in patients (51). Additionally, studies by Ewelina Dziurkowska and others highlight that individuals with SCZ often experience high levels of psychological and physiological stress, potentially leading to chronic cortisol secretion (52). Lies Langouche’s research further suggests that prolonged elevated cortisol levels can cause immune function suppression (53), exacerbating pulmonary inflammation and damage. Moreover, the comorbidity of SCZ with MDD complicates this scenario, as indicated by the mediation MR analysis in this study (Mediation effect: 60.45%, *P* = 6.98×10–^5^). Depressive states can lead to detrimental lifestyle changes, such as smoking and unhealthy dietary habits, thereby increasing the risk of COPD. An observational and MR mixed study by Bi Ran’s team provides consistent evidence for the association between MDD and an increased incidence of COPD (54). Overall, these psychophysiological health factors are crucial in the link between SCZ and COPD, suggesting the need to consider these factors in the treatment and management of SCZ to mitigate their impact on overall health.

This study highlights a significant causal association between genetically predicted SCZ and increased risk of COPD in EUR ancestry, which was not observed in EAS ancestries. The diverse genetic backgrounds across different racial groups may influence the association between SCZ and COPD. Additionally, varying economic conditions, levels of air pollution, cultural practices, substance use, and healthcare factors in different regions contribute to the observed discrepancies in results. Furthermore, considering that SCZ and autism spectrum disorder (ASD) share common genetic vulnerabilities and neurobiological mechanisms, such as immune dysregulation and altered neurodevelopmental processes (55), future research could benefit from exploring whether the mediators identified in the SCZ-COPD link, such as BMI, ASI, and MDD, also influence the risk of COPD in individuals with ASD. However, current epidemiological evidence on the association between ASD and COPD is limited, with studies primarily focused on asthma (56). Nevertheless, a recent MR analysis has established asthma as a risk factor for COPD (57), suggesting that respiratory diseases may have broader implications in neurodevelopmental disorders. This finding provides a new perspective for further investigating the potential causal relationships between ASD and COPD and emphasizes the need for deeper exploration of the connections between these two conditions.

This study presents several advantages. Firstly, it pioneers the use of MR analysis to delineate the causal relationship between SCZ and COPD, in contrast to previous observational studies. Secondly, the analysis across various ancestries provides a multifaceted perspective. Thirdly, robust methods were employed, as evidenced by all F-statistics exceeding 10, thus minimizing potential biases due to weak instrumental variables. Fourthly, we investigated the impact of confounding factors through MVMR analysis and subsequent mediation MR analysis confirmed partial mediation roles of BMI, MDD, and Age of ASI. Fifthly, the robustness of primary study results was enhanced by combining sensitivity analyses rooted in diverse statistical models with ‘leave-one-out’ approaches, thereby increasing the reliability of the evidence. However, this study has certain limitations. Firstly, due to the limited sample size and number of SNPs, we were unable to extract usable SNPs within the genome-wide significance threshold, which precluded the inclusion of Hispanic or Latin American (AMR) and African American or Afro-Caribbean (AFR) ancestries in the study. While it is possible to lower the threshold to include more SNPs, the resulting causal estimates would not be sufficiently robust, and the latest MR guidelines do not advocate for this approach (58). Therefore, future research should utilize larger, multi-ancestry GWAS datasets or individual-level data to explore the universality of the causal relationship between SCZ and COPD across populations. Secondly, the number of IVs available for analysis in the EAS ancestry was limited, as was the sample size, preventing the detection of sufficient statistical power. This may impact the inference of causal conclusions, and future studies with larger sample cohorts are required to confirm the results in EAS ancestry. In the mediation MR analysis, given the first MR assumption and the rate of sample overlap, appropriate GWAS data on inflammatory markers suitable for this study were not available. Therefore, future research is required to validate the significance of inflammatory markers. Lastly, our reliance on summary-level GWAS data precluded the possibility of subsequent subgroup analyses.

## Conclusion

This MR analysis established a causal relationship between genetic susceptibility to SCZ and an increased risk of COPD in individuals of European ancestry. Key mediators in this relationship include BMI, age of smoking initiation, and MDD, underscoring the importance of multifaceted interventions and preventive measures. However, no causal evidence was found in EAS ancestry, highlighting the need for larger-scale GWAS studies to verify the robustness of these results and to conduct additional analyses on the generalizability in AFR and AMR ancestries.

## Data availability statement

The original contributions presented in the study are included in the article/**Supplementary Material**. Further inquiries can be directed to the corresponding authors.

## Ethics statement

Ethical approval was not required for the study involving humans in accordance with the local legislation and institutional requirements. Written informed consent to participate in this study was not required from the participants or the participants’ legal guardians/next of kin in accordance with the national legislation and the institutional requirements.

## Author contributions

YN: Conceptualization, Data curation, Investigation, Methodology, Writing – original draft, Writing – review & editing. DZ: Formal analysis, Resources, Validation, Writing – original draft. WT: Data curation, Formal analysis, Writing – original draft. LX: Investigation, Methodology, Writing – original draft. XC: Supervision, Writing – original draft. YZ: Supervision, Visualization, Writing – review & editing. YF: Funding acquisition, Project administration, Software, Supervision, Writing – review & editing.
