# Supplementary material for: Body mass index, smoking behavior, and depression mediated the effects of schizophrenia on chronic obstructive pulmonary disease: trans-ethnic Mendelian-randomization analysis
Source: Front Psychiatry. 2024 May 22;15:1405107. doi: 10.3389/fpsyt.2024.1405107 (PMC11155452; doi:10.3389/fpsyt.2024.1405107)
Supplement: Supplementary file 2 [file DataSheet_2.docx]

**Supplementary Fig. S1** Genetic associations with SCZ (horizontal axis, standard deviation units) and with COPD (vertical axis, log odds ratios) at a genome- wide level of significance. (A) EUR-SCZ on COPD-GBMI (B) EUR-SCZ on COPD-FinnGen (C) EUR-SCZ on COPD-GBMI-RadialMR (D) EUR-SCZ on COPD-FinnGen-RadialMR (E) EAS-SCZ on COPD-GBMI (F) EAS-SCZ on COPD- Sakaue S (G) EUR-COPD on SCZ-GBMI (H) EUR-COPD on SCZ-GBMI





**Supplementary Fig. S2** Leave-one-out plot for MR analysis of SCZ on COPD. (A) EUR-SCZ on COPD-GBMI (B) EUR-SCZ on COPD-FinnGen (C) EUR-SCZ on COPD-GBMI-RadialMR (D) EUR-SCZ on COPD-FinnGen-RadialMR (E) EAS-SCZ on COPD-GBMI (F) EAS-SCZ on COPD- Sakaue S (G) EUR-COPD on SCZ-GBMI (H) EUR-COPD on SCZ-GBMI
